# Influence of Femoral Stem Geometry on Total Hip Replacement: A Comparison of Clinical Outcomes of a Straight and an Anatomical Uncemented Stem

**DOI:** 10.3390/jcm13216459

**Published:** 2024-10-28

**Authors:** Massimo Berdini, Roberto Procaccini, Gabriele Franco Zanoli, Andrea Faini, Aldo Verdenelli, Antonio Gigante

**Affiliations:** Department of Clinical and Molecular Sciences, Clinica Ortopedica dell’Adulto e Pediatrica, Università Politecnica delle Marche, 60126 Ancona, Italy; roberto.procaccini@sanita.marche.it (R.P.); gabriele.zanoli@ospedaliriuniti.marche.it (G.F.Z.); dr.faini.andrea@gmail.com (A.F.); averdenelli@alice.it (A.V.); a.p.gigante@staff.univpm.it (A.G.)

**Keywords:** total hip replacement, total hip arthroplasty, THA, THR, femoral stem geometry, straight stem, anatomical stem, uncemented stem

## Abstract

**Background/Objectives**: There are many aspects that may influence clinical outcomes in a total hip arthroplasty (THA). The influence of femoral stem on the clinical outcome of THA is probably underestimated in the literature. Our work aims to analyze how uncemented stem geometry (straight or anatomical) in THA might affect outcomes in clinical and radiographic terms. **Methods**: Over a period of 36 months, in a prospective-observational manner, we collected the results of THA secondary to osteoarthritis (OA) that met the inclusion criteria with the only variable being the straight or anatomical stem design in a single manufacturer. A total of 84 patients were selected and divided into two groups: group A, treated with straight stem (44 patients), and group B, treated with anatomical stem (40 patients). The assessment clinical tools were Harris Hip Score (HHS), Visual Analogue Scale (VAS), and Short Form Health Survey-36 (SF-36). Follow-up controls were at 6 months (T0), 12 months (T1), 24 months (T2), and 36 months (T3). **Results**: No statistically significant differences emerged between the two groups under analysis with VAS, SF-36, and HHS. At follow-up controls, eight patients (group A) and four patients (group B) showed anterior thigh pain. At T1, there were radiographic signs of aseptic loosening in two cases (group A) and one case (group B). In group A there were two cases of iatrogenic fracture, two cases of dislocation, one case of infection, and two cases of heterotopic ossification. **Conclusions**: The anatomical stem compared to the straight stem showed lower complication rates outcomes; the anatomical uncemented stem could be considered as a preferred first choice in THA compared to the straight stem.

## 1. Introduction

Total hip arthroplasty (THA) is widely regarded as one of the most successful orthopedic surgical procedures of the last century [1]. The main purpose of a hip arthroplasty is to achieve a stable, pain-free joint with full range of motion, to correct deformities enabling the patient to perform daily activities without significant impairment. Additionally, the procedure should be durable and not result in intolerance [2,3]. The most common indications for THA are primary and secondary osteoarthritis, with other indications being aseptic necrosis of the femoral head, rheumatic arthritis, or femoral neck fracture [4].

A substantial corpus of data can be found in the existing literature on total hip arthroplasty (THA), focusing on every aspect of this surgical procedure that may potentially influence its success or failure. Among other factors, surgical approaches [5,6], prosthetic material and geometry [7], implant geometry in relation to the patient’s hip anatomy [8,9], prosthetic components, and the bone–prosthesis interface have been the subject of extensive analysis [10]. The study of these and other aspects has led to continuous evolution of implants since Charnley’s concept of mid-20th century, resulting in significant advances in implant reliability, success, and durability [1,11,12].

The design of stem implants has evolved over time to achieve optimal bony integration, favorable tribological properties, minimal bone resorption, and preservation of hip geometry [13].

Over the past two decades, there has been a notable shift in surgical approach to total hip arthroplasty (THA) in the United States, with cementless femoral fixation becoming the preferred method over cemented implants. An analysis of data from global registries on cemented or cementless total hip arthroplasty (THA) reveals a preference for cemented femoral component fixation in most of cases, as reported by the Australian Orthopaedic Association (21.8%) and the National Joint Registry for England (63.4%). In contrast, in the United States, orthopedic surgeons have a predominantly cementless implant preference (93%) [14].

The choice between cemented and cementless fixation is not solely a matter of surgical technique, but also a decision with implications for patient outcomes. A paucity of literature exists regarding cemented versus uncemented femoral stem fixation in the context of total hip arthroplasty (THA) [15,16].

The choice option for cementless fixation of the femoral stems lies in several factors and may relate to reduced operative time, concerns about the physiological reaction to cement pressure, and the observed reduced risk of aseptic loosening [17,18].

Despite the abundance of literature on total hip arthroplasty (THA), there is a surprising lack of interest in the potential influence of stem geometry on clinical outcomes [19].

Our work aims to investigate whether there are differences in patient-reported outcome measures according to the geometry of the femoral stem in THA, straight or anatomical, with a 36 month follow-up. In addition to the clinical rating scales, the endpoints were the rate of thigh pain and the rate of iatrogenic fractures caused by the two stem types; the rate of aseptic loosening and the patient’s quality of life were selected as secondary end-points.

## 2. Materials and Methods

The study was designed as observational, cross-sectional without parallel cohort, and conducted over 36 months at the Clinica Ortopedica dell’Adulto e Pediatrica of the Azienda Ospedaliera “Ospedali Riuniti”–Ancona, Italy. The patients were enrolled over a one-year period, with a subsequent three-year follow-up phase.

The article’s writing followed the Strengthening the Reporting of Observational Studies in Epidemiology (STROBE) checklist (Supplementary Materials).

Patients were aged between 65 and 75 years, both genders, with a radiological finding of Dorr B femoral canal undergoing cementless hip arthroplasty due to OA, in a consecutive order.

Data were collected on surgeries performed by a single surgeon experienced in hip arthroplasty. The surgeon who performed the surgical treatment did not take part in data collection or analysis of the study. The choice of prosthetic implant was made by the surgeon according to patients’ specific characteristics and preoperative planning; the examinators responsible for collecting and processing the patients data were not involved at this stage.

After the surgical treatment, two expert investigators (both orthopedic surgeons, also mentioned as “examinators”) collected clinical data of the patients, surgical information, implant choice made by the surgeon, and inclusion and exclusion criteria. Patients recruiting was, thus, conducted ensuring independence between the surgeon and the examinators in a blinded pre- and postoperative manner.

The radiographical findings were examined by the same two examinators and subsequently checked by two radiology specialists, who reached their statements independently in a blinded way.

Patients who met the inclusion criteria were informed of the details of the study and asked to provide written consent to participate in the present study, including follow-up controls. The study was conducted in accordance with criteria set out in the Declaration of Helsinki [20].

All patients enrolled in the study underwent comprehensive clinical and radiological examinations at the study’s outset and then at scheduled follow-up times at 6 months post-discharge (T0) and at 12 months (T1), 24 months (T2), and 36 months (T3) after discharge time.

### 2.1. Exclusion Criteria

Exclusion criteria for patients in the study were identified as pain consistent with organic or neurological causes unrelated to hip replacement (i.e., radiculopathies and myopathies), current smoke habit, diabetes, and severe heart-related diseases. These patients were excluded because these pathologies may have a direct or indirect effect on the prosthetic osseointegration process or the rehabilitation pathway, and therefore represent a confounding factor for clinical outcome.

Patients that presented existing deformities, as documented in their medical history and/or identified through preoperative radiographic imaging, were excluded from the evaluation. These conditions included a receding acetabular roof (>40° using the pelvic teardrop reference), severe hip deformity or ankylosis of the hip, and dysmetria exceeding 1 cm. Additionally, patients with neurovascular deficits of the lower extremities were excluded.

### 2.2. Patient Management and Evaluation

Upon admission, each patient was evaluated by two examinators with extensive experience in the field of orthopedics and THA. The evaluation was based on three key factors: pain, overall health status, and hip function. The assessments were conducted with the following tools: Visual Analogue Scale (VAS) for the evaluation of pain, Harris Hip Score (HHS) as disability score related to the hip function, and Short Form Health Survey (SF-36) to assess both mental and physical status of each patient. The measurements were repeated at each designated follow-up interval for each patient.

Furthermore, the occurrence of anterior thigh pain was subjected to specific examination; we performed the anvil test to evaluate any clinical signs of prosthetic implant displacement.

Patients received standard preoperative antibiotic prophylaxis with cefazolin 2 g in the operating room 60 min before the surgical treatment and a postoperative cefazolin 2 g booster 12 h after surgery. Antithrombotic prophylaxis was administered to the patients in the form of low-molecular-weight heparin (LMWH) 4000 international units, with the initial dose administered 12 h after surgery and subsequently administered daily until the 30th postoperative day.

All patients underwent the placement of a bladder catheter in the operating room, which was subsequently removed 24 h after surgery.

The patient’s body temperature was monitored twice daily throughout the duration of their hospitalization.

From the postoperative day, patients were encouraged to engage in passive mobilization and trained on a physiotherapy program including exercises designed to aim hip articulation and weight bearing. The rehabilitation program started during the second postoperative day, with the use of crutches for a seven-day period, and subsequently progressed to autonomous weight-bearing. The rehabilitation program was conducted and developed under the guidance of a dedicated physiotherapy team.

The removal of stiches on the surgical wound was performed on the fifteenth postoperative day.

During clinical follow-up, each patient underwent examinations conducted by the same two operators for the entire evaluation period. The aim was to conduct an orthopedic clinical examination of the lower extremities. Furthermore, in addition to any possible dysmetria, the active range of motion (ROM) of the prosthetic hip was assessed in relation to flexion–extension, internal and external rotation, abduction, and adduction. Additionally, any evidence of thigh pain was investigated, which could indicate imperfect centering of the stem in the femoral canal, by performing the anvil test.

Pain registered by the examinator in the anterior and/or lateral thigh below the inguinal region was considered to be anterior thigh pain, according to the criteria stated by Barrack et al. [21]. The anvil test was considered positive if the patient felt pain in the hip region or in the proximal region of the thigh when the examinator struck the calcaneus with a fist, with the patient in the supine position and the hip flexed at mid-angle [22].

Furthermore, pain was evaluated in the affected limb using the Visual Analogue Scale (VAS) pain rating scale [23]. Two tests with clinical and functional scores were administered to the patients in order to assess their global health: the Short Form Health Survey-36 (SF-36) index for both domain of physical component summary (PCS) and mental component summary (MCS) (IQOLA SF-36 Italian Version 1.6) was assessed independently by the patient, while the HHS (Harris Hip Score) was administered by the clinician [24,25].

### 2.3. Surgical Management and Prosthetic Implant

The surgery was performed via postero-lateral access according to the Gibson–Moore technique, with the neck osteotomy carried out at the isthmus level and the extrarotator tendons reinserted.

The prosthetic hip implant under analysis in this study was manufactured by a single producer. The producer was Adler Ortho S.p.A. having Apta^®^ or Recta^®^ as femoral modular cementless stem with fixed bearing, with a Fixa Ti-Por^®^ acetabular cementless metal back implanted (without use of screws), a polyethylene liner, and a medium ceramic finished femoral head Biolox^®^ delta 36 mm with a neutral medium (0Y) 12/14 Modula^®^ femoral neck.

The sizes of the femoral stems were established through preoperative planning and subsequently validated intraoperatively, in accordance with the patient’s anatomical characteristics. During the surgical procedure, the established anatomical landmarks from the preoperative planning were used to restore the length of the lower limb undergoing surgery. This was performed in order to prevent the potential occurrence of iatrogenic dysmetria, resulting as surgical inaccuracy.

The manufacturer did not provide funding or influence the decision-making process considering the selection of the prosthetic implants used in these cases, nor financially supported this study.

All patients were placed under subfascial suction drainage at the surgical site, which was removed 24 h after surgery.

### 2.4. Radiological Evaluation

An X-ray of the pelvis in the antero-posterior projection and an X-ray of the surgically treated hip in the axial and antero-posterior projections were performed to each patient.

The study cohort consisted of patients exhibiting a type B Dorr femoral canal conformation as determined by preoperative antero-posterior pelvis and femoral X-rays [26].

The radiographical controls examined in this study were as follows: the preoperative radiographic control was conducted to determine the conformation of the femoral canal and the degree of hip arthrosis. Subsequent clinical and radiographic postoperative controls were performed as established by the follow-up schedule, up to 24 months post-surgery. The clinical evaluations were conducted by two experienced orthopedic surgeons, and the radiological evaluations were provided by two experienced radiologists in a blinded manner.

The following parameters were taken into account: position of the stem within the femoral canal, positioning of the prosthetic acetabulum, hip offset, presence of periprosthetic osteolysis, as determined by the Paprosky index parameters [27], and the existence of stress fractures and radiographical stress-shielding signs.

### 2.5. Statistical Analysis

The observed population was divided into two groups based on the morphology of the implanted stem: group A, treated with straight stem, and group B, treated with anatomical stem.

A statistical analysis was performed to determine the mean values of the data collected from the clinical tests, including those related to range of motion and test results (VAS, HHS, SF-36), of group A and group B.

The results of the descriptive parameters are reported in terms of mean and standard deviation.

The mean values of the results obtained from the two groups were compared over the follow-up period for each variable of interest.

We adopted a nonparametric test for independent samples, the Mann–Whitney U-test, to assess the presence of a similar or dissimilar distribution between the two groups (group A, group B) based on the values of a quantitative, at least ordinal variable.

The null hypothesis states that the distributions between the populations in question are equal. Conversely, if the distributions within the populations are found to differ, the null hypothesis is rejected.

The level of statistical significance of the Mann–Whitney U-test was established to be equal to or greater than 0.05, with a corresponding *p*-value of 0.05.

Levene’s test was utilized in conjunction with the *T*-test for equality of means to evaluate the extent of sample variance or dissimilarity. The assumption of equal or unequal variances was contingent upon the values as reported by the test. Differences in behavior between the values of interest at the specified times between the two populations was identified through the utilization of the *T*-test (means-equality test).

The *T*-test is a parametric statistical test analogous to the Mann–Whitney U-test. Its purpose is to ascertain whether the mean value of a distribution deviates significantly from a reference value. It was employed in conjunction with the Levene’s test to assess the variance of the populations under examination. The previous mentioned analyses enabled an evaluation of whether a statistically significant discrepancy existed between the mean values of the two samples in the comparable populations.

The radiographic evaluation was conducted using the company software Centricity Web (V3.0), developed by GE Medical Systems Information Technologies.

The data collection and compilation of the database and graphs were conducted using Microsoft Office 365 suite (Ver 2407, build 17380.20138), while SPSS software (IBM SPSS STATISTICS V27.0.1 IF026) was used for the statistical analysis.

## 3. Results

A total of 94 patients who underwent primary total hip arthroplasty with an OA diagnosis at the Clinica Ortopedica dell’Adulto e Pediatrica of the Azienda Ospedaliera “AOU Ospedali Riuniti”–Ancona-Italy during the one-year period were enrolled according to the specified criteria.

A total of 10 patients (10.6%) were lost to follow-up at the outpatient visit 6 months after surgery, of whom 6 had an anatomic stem and 4 had a straight stem implanted. These patients were excluded from the study.

**Figure 1:**
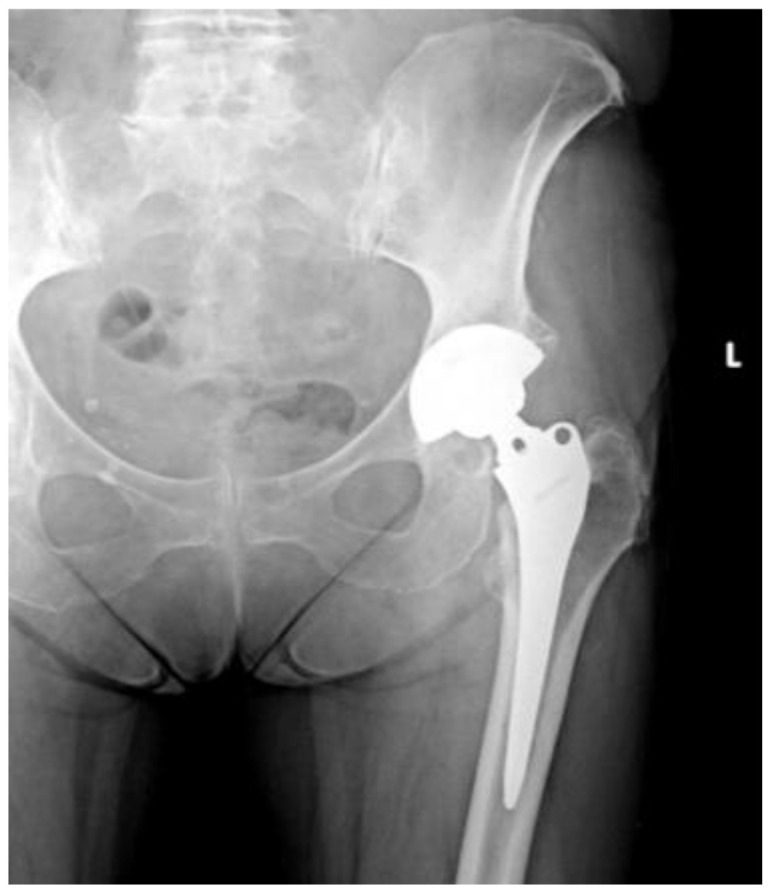
Antero-posterior X-rays view of a total hip arthroplasty with straight stem (group A).

**Figure 2:**
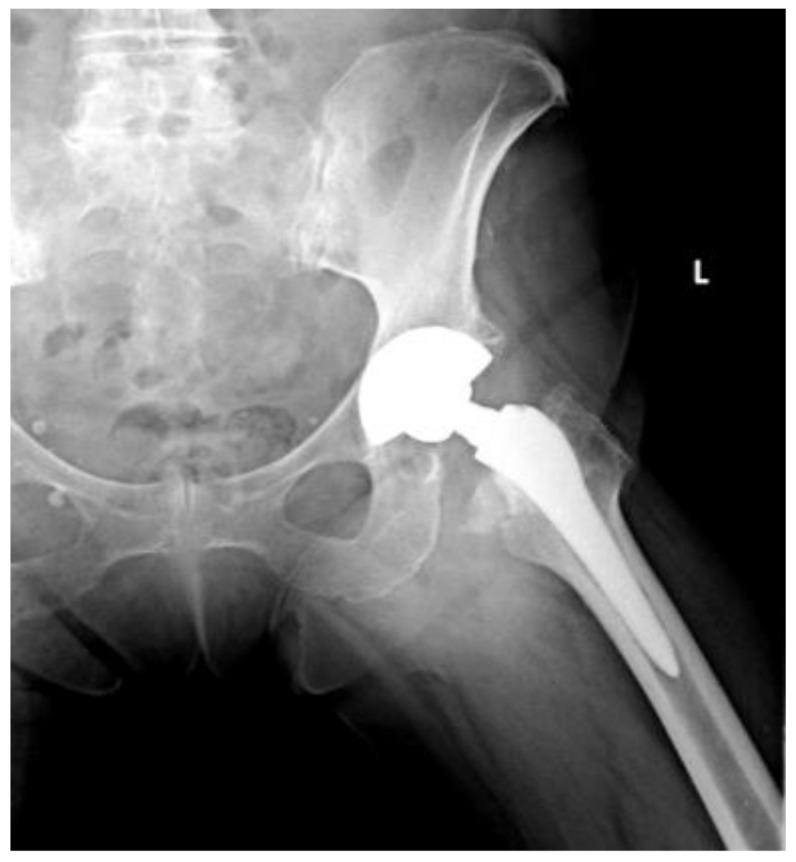
Axial X-rays view of a total hip arthroplasty with straight stem (group A).

**Figure 3:**
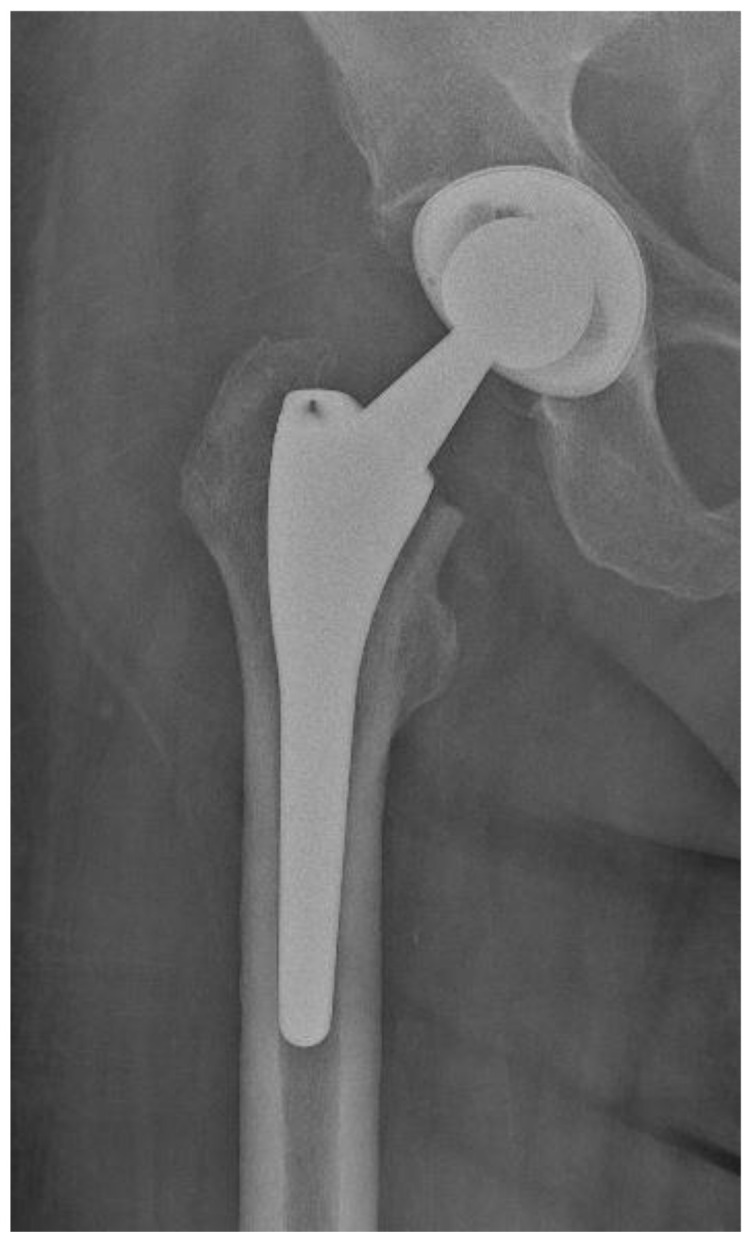
Antero-posterior X-rays view of a total hip arthroplasty with anatomical stem (group B).

**Figure 4:**
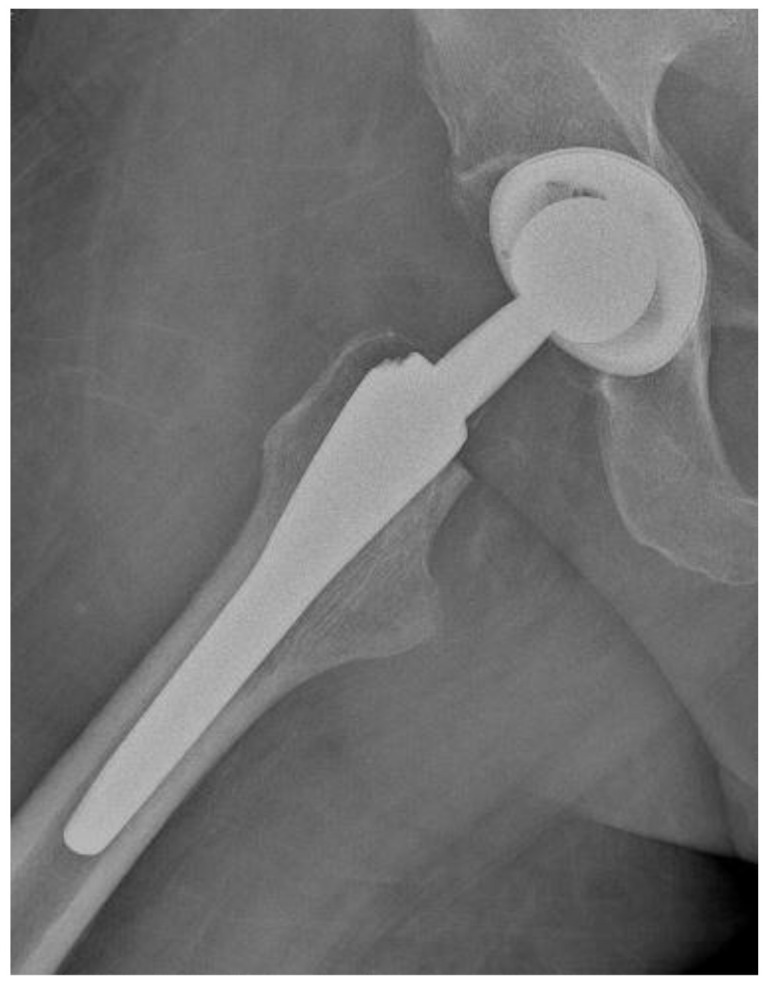
Axial X-rays view of a total hip arthroplasty with anatomical stem (group B).

Of the 84 patients enrolled in this study, 44 patients (52.4%) received a THA with a straight stem (group A) (Figure 1 and Figure 2) and 40 patients (47.6%) received a THA with an anatomical stem (group B) (Figure 3 and Figure 4).

At 6 months (T0), 1 year (T1), 2 years (T2), and 3 years (T3) post-surgery, these patients continued the established follow-up.

Analyzing the gender of the patients, group A consisted of 25 female patients (55.7%) and 19 male patients (44.3%) and group B had 23 female patients (56.4%) and 17 male patients (43.6%). The other patients’ demographics are shown in Table 1.

The results of the population distribution for the values considered, analyzed using the independent samples Mann–Whitney U-test, are shown in Table 2.

Considering the outcomes of the HHS and the SF-36 MCS and the SF-36 PCS, it was concluded that the two population groups were independent of each other and that the observed data were distributed normally. Thus, a parametric approach, based on the *T*-test for the comparison of means, was used for the statistical analysis.

The results of the two-sample *T*-test for equality of means yielded the following values, with a *p*-value = 0.05 are shown in Table 3.

The results of this test were analyzed to determine if there were any significant difference between the two groups in terms of averages of parameters under examination.

The results of the *T*-test showed no statistically significant differences considering VAS for each domain of SF-36 PCS-MCS and HHS.

Pain, as assessed by means of the VAS, and quality of life of the recruited patients, as assessed by means of the SF-36 scoring system, at T0, T1, T2, and T3 showed no significant difference in anatomical versus straight stem implant (Table 4).

The values of the SF-36 showed an upward curve after the sixth month (T1) and reached a plateau after one year (T2) of follow-up (Table 5).

Parameters used to evaluate patient’s quality of life in the postoperative period show a consistent pattern, exhibiting a gradual improvement from the sixth month onward.

No iatrogenic fractures were observed in group A. Meanwhile, in group B, two cases of greater trochanter fracture (classified as type A according to the Vancouver classification) were observed, affecting two female subjects. These were treated promptly with the application of metal cerclages [28].

The analysis showed no significant differences in HHS values between the two stem types under investigation (Table 6).

Data from the HHS evaluations demonstrate a growth trend following the sixth month of observation (T1), subsequently reaching a steady state after a period of one year (T2) of monitoring.

The analysis of the data concerning the clinical outcome of active hip ROM revealed no statistically significant differences between the two groups.

Nevertheless, it is observed that the THA brings the restoration of optimal hip mobility in both groups within the initial six-month period. Stable values are achieved at the six-month follow-up with an onwards trend. The results between the two groups are comparable in terms of flexion, extension, abduction, and adduction degrees. In order to avoid risk of dislocation of the prosthesis, hip internal rotation was not tested. The values recorded by group B (anatomical stem) were higher than those achieved by group A (straight stem) at all follow-up times in terms of active external rotation degrees of the hip (Table 7 and Table 8).

Radiographic evidence of aseptic loosening was identified in three male subjects within 12 months of surgery, of which two cases were observed in group A with a straight stem (Paprosky 2) and one case in group B with an anatomical stem (Paprosky 2). Prosthetic surgical revision was performed in all cases.

No differences in periprosthetic secondary remodeling of the bone were observed in radiographic examination between the two groups. Furthermore, no stress-shielding or periprosthetic cortical bone rarefaction was reported in either group.

From the sixth postoperative month onwards, 12 cases (8 patients (6 female and 2 male) in group A and 4 patients (3 female and 1 male) in group B) were observed to present with anterior thigh pain. The referred pain gradually disappeared over time, reaching complete resolution at 12 months, without performing any other treatment.

In addition, two episodes of traumatic hip dislocations were observed in the fourth week following surgery, and seven cases of dysmetria exceeding 1 cm were documented (three group A and four group B prostheses). The maximum value observed for dysmetria was 2.0 cm. The mean value was 1.3 cm +/− 0.7 cm. Furthermore, two cases of heterotopic ossification of the hip (Brooker II) [29] and one case of prosthetic infection in group A that underwent prosthetic revision using the two-step method were also identified.

## 4. Discussion

One of the most successful orthopedic surgical procedures of the last century is total hip arthroplasty (THA) [1]. The objective of a hip arthroplasty is to achieve a stable, pain-free joint with optimal articulation, to correct deformities, enabling the patient to perform daily activities without significant impairment. Additionally, the procedure should be durable and not result in intolerance [2,3]. The most common indications for THA are primary and secondary osteoarthritis, other indication but with a lesser prevalence aseptic necrosis of the femoral head, and rheumatic arthritis or femoral neck fracture [4].

In a total hip arthroplasty (THA), both the acetabulum and the femoral head are replaced. This procedure requires the insertion of a metal stem into the femur, which is then associated with a modular head that articulates with a nonmetal insert and a metal back artificial cup, which constitutes the acetabular component. Among the various combinations of bearing surfaces that have been used, a metal femoral head and a polyethylene acetabular cup have become the most widely used since the introduction of the low-friction concept by Charnley [30,31,32].

The choice of a cementless fixation of the femoral stems lies in several factors and may relate to reduced operative time, concerns about the physiological reaction to cement pressure, which may lead the patient to develop threatening complications, and the observed reduced risk of early or late aseptic loosening [17,18].

The most important objective in the surgical treatment of uncemented THA is to achieve a successful osseointegration of press-fit prosthetic components. This objective lies in three core concepts: the initial stability of the implant, which relies on the primary fit at the bone–prosthesis interface; the medium-term biological fixation of the prosthesis that lies on the processes of direct osseointegration with the bone (bone ingrowth and ongrowth); a balanced distribution of load across the femoral implant, which depends on the positioning of the prosthetic implant and its geometric conformation, and is necessary for long-term durability and survival of the prosthetic implant [33,34,35,36].

These factors not only impact bone tissue but also, more broadly, directly influence the performance of prosthetic replacement, which can be evaluated according to clinical outcomes [37,38].

A substantial corpus of data can be found in the existing literature on total hip arthroplasty (THA), which analyzes every aspect of this surgical procedure that may potentially influence its success or failure. Among other factors, surgical approaches [5,6], prosthetic material and geometry [7], implant geometry in relation to the patient’s hip anatomy [8,9], prosthetic mobility interfaces, and the bone–prosthesis interface have been the subject of extensive analysis [10]. The study of these and other aspects has led to a constant evolution of implants since Charnley’s concept of mid-20th century, resulting in significant advances in implant reliability, success, and durability [1,11,12].

The design of stem implants has evolved over time to achieve optimal bony integration, favorable tribological properties, minimal bone resorption, and preservation of hip geometry. In order to facilitate optimal reconstruction, different manufacturers offer a range of stem types with varying dimensions, lengths, shapes, offsets, and depositions. The choice of a specific implant is not solely based on the femoral characteristics and the planned surgical treatment, but also on implant costs and availability, familiarity of surgical team with instruments and implants, ease of use, and surgeon preferences [13].

Cementless femoral stems have been the subject of classification proposed by Khanuja et al. [39]. This classification system was subsequently modified by Kheir et al. to include short stems [40]. This classification identifies seven implant types, further subdivided by stem geometry, cross-sectional characteristics, and surface characteristics. Although these classification systems allow a structured discussion of cementless stem outcomes, their specificity in classifying stems based on stem geometry, cross-sectional properties, surface features, and length make the classification of various new and even established stems difficult.

Currently, there is not a clear classification system that allows comparison of how similar femoral stem design features affect clinical outcomes and survivability. Consequently, most of the current literature on femoral stem design is limited to analyzing individual femoral components [41].

Following these studies, Radaelli et al. proposed another classification to help standardize and compare uncemented femoral stems [33].

The stems involved in our study were uncemented femoral stems produced by a single manufacturer. In order to provide a classification of implanted stems in our work, the Recta^®^ stem, which is a straight femoral stem, could be included in group B (B1) as defined by Radaelli et al. This type of stem shows a rectangular cross-section, gradually tapered, engaging primarily in the meta-diaphysis. The Apta^®^ stem, which is an anatomical femoral stem, could be included in group C (C2). This type of stem is designed to match the femoral canal geometry, specifically the posterior kink in the femoral metaphysis. The objective is to achieve proximal metaphyseal stability with full engagement of the femoral cortex, thereby ensuring primary fit and rotational stability. These implants are wide in the proximal anterior dimension and are side-specific [33].

The choice between straight and anatomical femoral stems on THA is reliant upon a number of factors, including the morphology of the femur [40,41]. In our study, we elected the Dorr’s Type B of the femoral canal due to its high prevalence (85%) [26] and suitability for the insertion of both types of implants.

The impact of these implants on periprosthetic femoral bone remodeling has been investigated through the implementation of a range of study designs [42,43]. Furthermore, only a restricted number of studies have directly compared the conformation of anatomical versus straight stems by analyzing radiographic parameters, implant survival itself, or the possible incidence of complications [44,45,46], but not focusing on the possible influence of femoral stem prosthetic components on the clinical outcome.

The analysis of the clinical–radiographic parameters of our study shows a higher incidence of thigh-pain-related symptoms in group A (straight stem) (8 cases) compared to group B (anatomical stem) (4 cases). This symptom gradually disappeared over time, reaching complete resolution at 12 months, without performing any other treatment [47]. The aspect of anterior thigh pain is widely discussed in the literature and is often referred to as the femoral component at its interface with the cortical bone [48,49,50]. In a recent work made by Kato et al. focusing on pain developed by different contact on the cortical bone between a tapered-wedge femoral stem and a fit and fill femoral stem, it is reported that the incidence of pain, included the thigh pain, is higher in the tapered-wedge stem than in the fit and fill stems [50]. Studies have reported that anterior thigh pain is either absent or at least tolerable, but all reported data were from the analysis of short femoral stems [45,51]. In some studies, the cause is reported as oversizing of the femoral component [48,52,53,54,55,56]. Our data appear to be consistent with the latter evidence on pain trends and pain distribution between two types of femoral implant. The reduced number of cases observed in group B could be attributed to the morphology of the anatomical femoral stem, which exhibits a predominantly metaphyseal grip and may, therefore, exert less stress on the diaphyseal bony corticals.

Considering iatrogenic fractures, the incidence of this type of complication in the literature is approximately 1% of cases in THA with the implant of straight stems. Such fractures are frequently diaphyseal and are accountable to the pursuit of optimal press-fit in a stem with a rectangular cross-section [53,57,58,59]. Our data do not show any iatrogenic fractures in the subjects belonging to group A. In group B, only two cases of a fracture of the greater trochanter with a stable femoral stem were treated intraoperatively with metal cerclages. It can be reasonably assumed that this kind of complication is the result of the conformation of the anatomically profiled stem, which, having a greater proximal diameter than the straight stem, may lead to this kind of complication when seeking the optimal press-fit during implant operations. The pursuit of an optimal press-fit could lead the orthopedic surgeon accidentally oversizing the implanted stem, thereby increasing the risk of iatrogenic fractures of the diaphysis or trochanter and a heightened likelihood of residual tip pain. Notwithstanding the potential complications, an undersized stem would entail a considerable risk of aseptic loosening.

Recent studies have shown that aseptic loosening represents the most prevalent cause of revision of THA. Revisions due to infection, fracture, and instability occurred more frequently during the early post-THA period after primary THA. Revisions due to osteolysis, instability, and acetabular wear have increased in recent years [60].

The literature on this topic suggests that undersizing of the femoral stem is the first cause of aseptic loosening. The undersizing of the femoral stem component may be associated with a lack of confidence in achieving a strong press-fit during the implant procedure, potentially due to concerns about the potential for iatrogenic fractures [60,61,62].

In this regard, although our follow-up period was relatively short, it is interesting to point out that the radiographic aspects of early migration of the femoral stem could show a high predictive capacity for late prosthetic failure. As Kroell et al. emphasize in their work, early aseptic loosening can be interpreted as a consequence of primary instability due to poor technique or implant design [63].

In examining the dataset of our work, it was observed that instances of aseptic loosening occurred in a total of three cases, two in group A and one in group B. The relatively small sample size of our study groups probably affected this outcome, which did not show significant differences between the two groups.

To the best of our knowledge, there are no studies that directly compare the two types of stems being analyzed in our study. However, there are various articles that analyze this type of complication. In the work of Alexander et al., where complications related to an uncemented straight-type femoral stem (tapered stem) are analyzed, an incidence of 1.3% over a follow-up time of 25 years is reported [64]. Another study points out that single-wedge femoral stem geometry shows a higher incidence of complications related to aseptic loosening in comparison to stems with a double-wedge design [65]. These data, despite examining a different stem type, appear to be consistent with our findings.

In analyzing the results deriving from the clinical tests, although there were no statistically significant differences between the two groups under examination, we can note that pain (analyzed with the VAS scale), the functional recovery of the surgically treated hip (analyzed with the HHS), and the recovery of the global state of health (analyzed with SF-36) showed a significant increase in the postoperative period thanks to the surgical treatment performed, which reached a peak one year after surgery and then continued according to a stable trend at the next follow-up times without any decrease.

These findings confirm what has been reported in the literature, namely, that THA has a significant positive impact on the recovery of function of the surgically treated joint and the restoration of a satisfactory quality of life [34,45,66].

As reported in the literature, the assessment of not only clinical scores, but also the simple recording of degrees of hip motion observed by the examiner during the postoperative clinical examination in vivo, can be a highly valuable predictive indicator for the outcome of THA [67].

Despite the absence of statistically significant differences between the two groups in our work, both demonstrated a satisfactory recovery of hip range of motion at values considered average in comparison to the results reported in the literature (flexion 114–90°, abduction 24–16°, external rotation 19–11°) [67].

Patients treated with anatomical stems demonstrated a trend toward faster recovery of flexion, extension, abduction, and external rotation degrees of range of motion compared to patients treated with a straight stem, although this observation lacks statistical significance. These last findings are not comparable with the existing literature.

Based on these observations, it can be hypothesized that the anatomical stem may have a positive influence on the functional recovery of the hip. This is indicated by the faster recovery at higher degrees of joint motion compared to the group with the straight stem.

The data could also suggest that in relation to a fixed size of femoral head, which typically determines the range of motion of the hip, the uncemented anatomical femoral stem could exhibit a more favorable effect on hip extrarotation postoperatively than a straight stem. We can hypothesize that this aspect should be attributed to the femoral stem geometry.

Further research is required to gain more comprehensive understanding of this topic.

To the best of our knowledge, no other studies have compared the clinical outcomes of total hip arthroplasty (THA) focusing exclusively on the variable of uncemented femoral stem geometry.

Many studies in the literature have analyzed various aspects of uncemented femoral stem geometries of THA and their effects from a radiographic point of view [46,68] on possible early and late complications [3,59] and their results in terms of implant survival [51]. In our opinion, one of the fundamental aspects of the outcome of THA surgery lies in the ability to restore a functional, pain-free, and durable joint to the patient [2]. A perhaps underestimated aspect, in our opinion, lies in the influence that the femoral component of THA can have on the overall outcome of surgery and the patient’s perceived overall satisfaction.

Although having no statistical relevance, our study seems to suggest a difference between anatomical and straight femoral stem choice in terms of both PROMs and active range of motion, especially in the short term, given the lower incidence of anterior thigh pain and higher degree of motion in external rotation in group B compared to group A between 6 and 12 months postoperatively.

This study also shows how this trend tends to vanish in the long term (after 12 months follow-up), pointing out that the stem’s choice might be more relevant in the early stage of the rehabilitation process, rather than in the advanced phase of recovery and osseointegration of the THA implant.

Our study has several limitations. Firstly, the sample size of the population under examination was relatively small and the results obtained did not reach statistical significance in many fields. It will be necessary to extend this study by enlarging the sample under investigation in order to be able to carry out a more reliable analysis of the parameters under evaluation. It would be of interest to include not only a larger sample of the test population, but also to introduce analyses of factors that were excluded from our study (e.g., very frequent comorbidities such as smoking, heart disease, etc.) in order to assess whether these aspects have an influence on the clinical outcome of the THA result.

This study was primarily clinical in methodology. It would be interesting for future studies to introduce other clinical tools capable of assessing the clinical progress of the implant performed and its impact on the functional recovery and overall health of the patient. With the aim of reducing potential sources of bias, we limited the number of examiners involved in data collection, we included the data from a single surgeon performing THA, and we selected a fixed combination for the cementless prosthetic implant. Furthermore, X-ray imaging was employed only for the initial diagnosis and to evaluate potential macroscopic complications, without pursuing additional and more comprehensive analyses utilizing second-level radiological techniques, such as computed tomography (CT). It would be interesting and could add value to future studies to add more precise radiographical exams (i.e., using CT scan) and use radiographical scores to evaluate the influence of the uncemented femoral stem on the bone tissue, as this may have a clinical correlation on the outcome of total hip arthroplasty (THA). The follow-up period was relatively brief. The decision to end the follow-up time of our patients at 36 months may have resulted in the exclusion of potential complications that could have arisen later and, thus, were not recorded.

Once again, considering the trend shown in our study might be helpful to develop further investigations, with a longer follow-up and with possibly larger samples, in order to catch any possible and relevant clinical difference so that a deeper understanding of the role of stem geometry in a successful THA might be achieved.

## 5. Conclusions

Total hip arthroplasty has become one of the most successful orthopedic surgical procedures in recent years. Patients who undergo this type of surgery for chronic degenerative joint disease, such as arthrosis, have the potential to regain full joint function and an excellent quality of life.

The success and spread of this surgical treatment have generated considerable interest within the scientific community considering all aspects of total hip arthroplasty.

In the available literature, possible key factors guiding the choice of prosthetic components remain a topic with considerable variability. This variability is often attributed to tradition, orthopedic surgeon preferences, and constant evolution of materials.

The objective of our study is to draw attention to the differences in the selection of the uncemented stem component in hip replacement surgery considering clinical outcomes.

Examining the femoral uncemented stems, no significant differences were found regarding the overall success of the surgery and the quality of life of the patients included in the study. However, a higher incidence of thigh pain and aseptic loosening was observed in straight stem compared to anatomical stem. All cases of iatrogenic fracture were registered in anatomical stem implants.

An evaluation of the range of motion recovery following arthroplasty surgery reveals that the mean values for flexion and external rotation are higher in the anatomical stems, although this difference is not statistically significant when compared to the straight stems. Nevertheless, the recovery of the hip joint is already satisfactory at six months post-surgery and remains at an excellent level one year later, with both implants exhibiting comparable trends in the recovery curve.

Based on these data, we can, therefore, suggest that a prosthetic implant with an anatomical stem could always be considered as the first choice in total hip arthroplasty in Dorr B femur morphology types, although further studies with larger population examined should confirm our results. The straight stem remains a viable and reliable alternative, supported by a substantial body of evidence.

## Figures and Tables

**Table 1:** Patients’ demographics. The values of Harris Hip Score (HHS), Short Form Health Survey (SF-36) for Mental Component Summary (MCS), and Physical Component Summary (PCS) were recorded preoperatively.

Variable	Value	
	Group A	Group B
Gender Male/Female	19/25	17/23
Age (y)	69 ± 8.3	71 ± 7.6
BMI (kg/m^2^)	24.5 ± 2.8	24.1 ± 3.3
HHS	62 ± 4.8	63 ± 3
VAS	7.2 ± 1.8	7.5 ± 1.5
SF-36 MCS	30 ± 8	30 ± 8
SF-36 PCS	27.8 ± 9	28 ± 9

**Table 2:** Results of the Mann–Whitney U-test for values Harris Hip Score (HHS), Short Form Health Survey (SF-36) for Mental Component Summary (MCS), and Physical Component Summary (PCS).

MANN–WHITNEY U-TEST			
	HHS	SF-36 MCS	SF-36 PCS
Follow-Up	*p*-Value	*p*-Value	*p*-Value
T0	0.117	0.615	0.959
T1	0.284	0.163	0.101
T2	0.456	0.461	0.224
T3	0.144	0.111	0.324

**Table 3:** Results of the *T*-test (means-comparison test) for values Harris Hip Score (HHS), Short Form Health Survey (SF-36) for Mental Component Summary (MCS), and Physical Component Summary (PCS).

*T*-TEST			
	HHS	SF-36 MCS	SF-36 PCS
Follow-Up	*p*-Value	*p*-Value	*p*-Value
T0	0.005	0.049	0.042
T1	0.000	0.042	0.005
T2	0.001	0.035	0.009
T3	0.001	0.013	0.012

**Table 4:** Comparison of results of means and standard deviation (SD) values of Visual Analogue Scale (VAS) between group A (straight stem) and group B (anatomical stem) at follow-up controls: 6 months after surgical treatment (T0), 12 months (T1), 24 months (T2), and 36 months (T3).

Visual Analogue Scale (VAS): Means Value				
	Group A	SD	Group B	SD
T0	4.2	*1*	4.5	*1*
T1	2.3	*2*.*2*	1.8	*0.8*
T2	1.6	*2*.*3*	1.1	*0.3*
T3	1.2	*2*.*1*	0.6	*0.3*

**Table 5:** Comparison of results of means and standard deviation (SD) values of Short Form Health Survey–36 (SF-36) between group A (straight stem) and group B (anatomical stem) divided into Mental Component Summary (MCS) and Physical Component Summary (PCS) at follow-up controls: 6 months after surgical treatment (T0), 12 months (T1), 24 months (T2), and 36 months (T3).

Short Form Health Survey-36: Means Value								
Mental Component Summary (MCS)				Physical Component Summary (PCS)				
	Group A	SD	Group B	SD	Group A	SD	Group B	SD
T0	37.5	*4.9*	37.5	*4.7*	33.5	*7.4*	33.7	*6.9*
T1	45.3	*10.4*	47.3	*8.7*	40	*8.2*	41.6	*9.7*
T2	46.8	*11.2*	48.7	*9.5*	42.7	*8.1*	43.1	*9.8*
T3	45.8	*11.4*	46.7	*10.1*	42.9	*7.6*	43.1	*9.9*

**Table 6:** Comparison of results of means and standard deviation (SD) value of Harris Hip Score (HHS) between group A (straight stem) and group B (anatomical stem) at follow-up controls: 6 months after surgical treatment (T0), 12 months (T1), 24 months (T2), and 36 months (T3).

Harris Hip Score (HHS): Means Value				
	Group A	SD	Group B	SD
T0	70.8	*4.9*	70.4	*2.4*
T1	84.4	*8.2*	85.6	*4.5*
T2	91.1	*5.0*	92.7	*4.2*
T3	91.7	*4.2*	93.5	*3.7*

**Table 7:** Results of means and standard deviation of the value recorded in clinical analysis of active range of motion of the hip treated with THA. Results of group A (straight stem) at follow-up controls: 6 months after surgical treatment (T0), 12 months (T1), 24 months (T2), and 36 months (T3).

Means of Clinical Values of the Hip Range of Motion: Group A										
	**Flexion**	SD	**Extension**	SD	**Abduction**	SD	**Adduction**	SD	**Extrarotation**	SD
T0	83.4	*11.2*	6	*2.1*	28	*6*	10	*2*	18.8	*10.4*
T1	85	*10.8*	8.6	*1.8*	30.6	*5.7*	12.6	*2.5*	30.1	*9.1*
T2	93.2	*10*	10	*1.2*	32.6	*5.5*	12.9	*3.1*	31.8	*8.6*
T3	100	*9.4*	12.8	*1.6*	32.5	*5.4*	12.5	*3*	36.8	*9.4*

**Table 8:** Results of means and standard deviation of the value recorded in clinical analysis of active range of motion of the hip treated with THA. Results of group B (anatomical stem) at follow-up controls: 6 months after surgical treatment (T0), 12 months (T1), 24 months (T2), and 36 months (T3).

Means of Clinical Values of the Hip Range of Motion: Group B										
	**Flexion**	SD	**Extension**	SD	**Abduction**	SD	**Adduction**	SD	**Extrarotation**	SD
T0	91.5	*12.5*	5.6	*3.3*	30.9	*6.5*	9	*4.2*	28	*11.8*
T1	93.9	*11.4*	10.2	*2.1*	35	*6*	10.2	*3.8*	42.7	*6.4*
T2	102	*8.9*	13.3	*1.8*	37.8	*4.2*	10.5	*3.1*	45	*3.6*
T3	105.8	*6.4*	14.5	*0.4*	38.7	*2.3*	9.6	*4.3*	44.6	*4.4*

## Data Availability

For the present study, data are unavailable due to privacy or ethical restrictions.

## Supplementary Materials

The following supporting information can be downloaded at: https://www.mdpi.com/article/10.3390/jcm13216459/s1.
